# Congenital Median Upper Lip Fistula

**Published:** 2012-06-01

**Authors:** Sajad Ahmad Salati, Bandar al Aithan

**Affiliations:** Assistant Professor of Surgery, College of Medicine, Qassim University, KSA; Consultant Plastic Surgeon, King Fahad Medical City, Riyadh, Kingdom of Saudi Arabia

**Keywords:** Fistula, Congenital, Median, Lip

## Abstract

Congenital median upper lip fistula (MULF) is an extremely rare condition resulting from abnormal fusion of embryologic structures. We present a new case of congenital medial upper lip fistula located in the midline of the philtrum of a 6 year old girl.

## INTRODUCTION

Congenital sinuses and fistulae of the lip are uncommon malformations [1,2]. Median upper lip fistulae (MULF) are extremely rare variant of congenital facial malformations and till date less than hundred cases have been reported in literature. The one opening of the fistulae are in the philtrum and other near the frenulum as compared to more common upper lip sinus which has a single opening on the philtrum that has a blind end. Multiple hypotheses have been proposed to explain the formation of these fistulae. We report a case of isolated congenital median upper lip fistula in a 6 year old girl.

## CASE REPORT

A six-year-old female child was brought with the history of swelling of upper lip with discharge. The swelling was noted shortly after birth. It discharged thick material periodically. The parents had learned to decrease the size of the swollen lip by squeezing and letting the secretions out. On examination, the patient had a palpable lump over philtrum of upper lip about 8mm x 6 mm with two minute openings, one (cutaneous) on the midline of philtrum (Fig. 1A) and another (mucosal) towards the right of frenulum (Fig. 1B). On applying gentle pressure, thick creamy sebaceous material could be expressed from cutaneous opening. The patient was operated. After partial expression of sebaceous material, 0.2cc of 0.5% methylene blue was injected (Fig. 2A). The dissection was carried out from mucosal side (Fig. 2B) and the whole of the fistula was excised. On the philtrum, a small ellipse of skin around the cutaneous mouth of fistula was excised to ensure complete excision. Histopathological examination of the specimen showed features of fibrous fistulous tract with central cystic dilatation. The inner lining comprised of stratified squamous epithelial and contained dermal appendages including sebaceous and sweat glands. At one year of follow-up, there was an inconspicuous scar in the middle of philtrum (Fig. 3).

**Figure:**
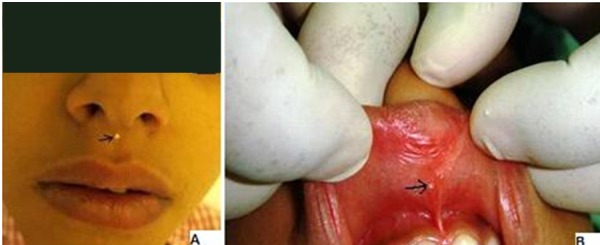
Figure 1: (A) Cutaneous opening of upper lip fistula over midline of philtrum with thick secretion- black arrow, (B) Oral mucosal opening of upper lip fistula adjacent to frenulum- black arrow.

**Figure:**
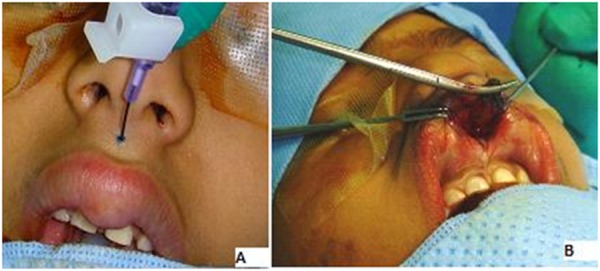
Figure 2: (A) Methylene blue injection into upper lip fistula. (B) Dissection of upper lip fistula through mucosal approach.

**Figure:**
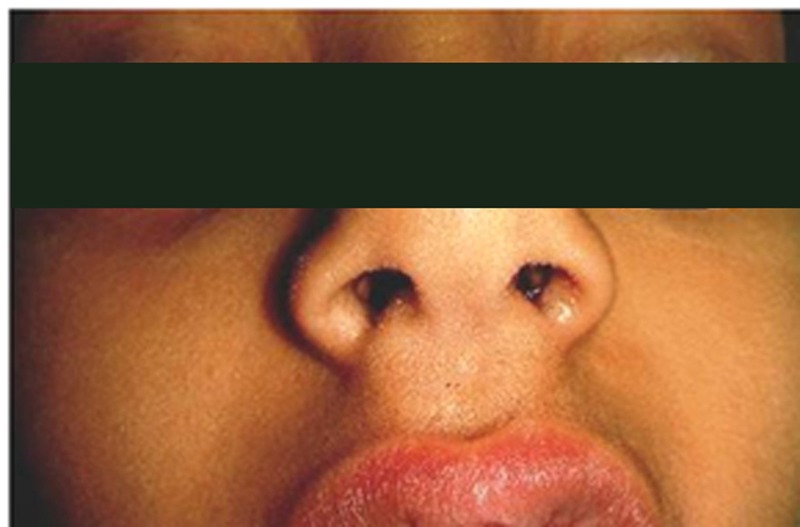
Figure 3: Follow-up picture with barely visible scar over philtrum (after 1 year).

## DISCUSSION

Lannelongue and Menard were the first to report this rare entity in 1891 [3] and since then less than 100 cases have been recorded in literature. The pathogenesis of midline upper lip fistulae is still debatable and a number of hypotheses have been proposed on this issue. Some have proposed that the early or abnormal epithelial inclusion events may occur in the medial fusion area during formation of the intermaxillary process [2,4,5] while others have suggested abnormal fusion of facial prominences or merging of mesoblasts [6]. The upper lip fistulae are seldom associated with other malformations [7]; however if such co-anomalies occur, they are midline malformations, such as double frenulum, medial cleft, nasal dermoid or hypertelorism [8].

Management comprises of complete surgical excision; incomplete excision leads to recurrences and ultimately cosmetic deformities. Based on our previous experiences of management of fistulae in other areas of body, we injected 0.5% methylene blue through the cutaneous mouth of fistula to facilitate the complete excision as entry into the fistulous tract would have stained the tissues and served as indicator of wrong plane of dissection.

## Footnotes

**Source of Support:** Nil

**Conflict of Interest:** None declared
